# Extrafloral nectar as entrée and elaiosomes as main course for ant visitors to a fireprone, mediterranean‐climate shrub

**DOI:** 10.1002/ece3.9500

**Published:** 2022-11-08

**Authors:** Byron B. Lamont, James Grey

**Affiliations:** ^1^ Ecology Section, School of Life and Molecular Sciences Curtin University Perth Western Australia Australia; ^2^ Present address: Assurance Systems North Sydney New South Wales Australia

**Keywords:** Adenanthos, elaiosome, extrafloral nectary, Fabaceae, Iridomyrmex, Mediterranean climate, myrmecochory, nonsprouter, parasitoid, Proteaceae, Rhytidoponera

## Abstract

Thousands of plants produce both extrafloral nectaries (EFNs) on their leaves and nutrient‐rich appendages on their diaspores (elaiosomes). Although their individual ecology is well‐known, any possible functional link between these structures has almost always been ignored. Here, we recognized their co‐presence in the shrub, *Adenanthos cygnorum* (Proteaceae), and studied their function and interaction. We observed that the same ants frequently visit both structures, seeds are attractive to vertebrate granivores but are released into a leafy cup from where they are harvested by ants and taken to their nests, from which seeds, lacking elaiosomes, germinate after fire. We showed that juvenile plants do not produce EFNs and are not visited by ants. We conclude that EFNs are not just an indirect adaptation to minimize herbivory via aggressive ant visitors (the role of a minority) but specifically enhance reproductive success in two ways: First, by inducing ants to visit the plant as a reliable food source throughout the year. Second, by promoting discovery of the seasonally available, elaiosome‐bearing seeds for transport to their nests (the majority of visitors), so avoiding the risk of granivory should seeds instead fall to the ground. Parasitoid wasps play a supporting role in controlling the main insect herbivore whose larvae devour the reproductive apices. Thus, the EFN‐elaiosome relationship has three components that enhance species fitness: foliage protection, seed transport, and granivore escape. A similar system has been described only once before (in an unrelated biome) and, consistent with the objectives of ecology as an integrative science, deserves wider study.

## Graphical abstract

Thousands of flowering plants produce both extrafloral nectaries on their leaves and seeds with nutrient‐rich appendages. Their possible interaction was examined in an Australian shrub here and shown that their co‐presence serves to attract ants to the plant throughout the year that then take the seeds from the plant to their nests during the flowering season rather than allowing the seeds to fall to the ground where they are likely to be consumed by birds and rodents.

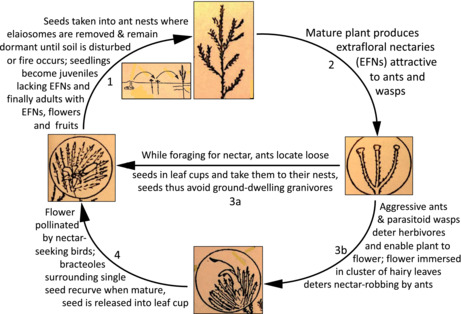

## 1 INTRODUCTION

It is intriguing that numerous plant families possess species that produce both extrafloral nectaries (EFNs) on their leaves as well as nutrient‐rich appendages attached to their diaspores (elaiosomes). Top of the list is the major family Fabaceae where they are co‐present in perhaps 50% of species, approaching universality in the huge genus *Acacia ss*, with 1100 species (Baker et al., 1978, González‐Teuber & Heil, 2009; Marazzi et al., 2019; Pausas & Lamont, 2022). Other families with their co‐presence include Euphorbiaceae, Polygalaceae, Lamiaceae, Proteaceae, Malvaceae, and Passifloraceae (Christian & Stanton, 2004; Dutton et al., 2016; Eriksen & Persson, 2007; Forest et al., 2007; Groom & Lamont, 2015; Krawczyk & Głowacka, 2015; Lengyel et al., 2010; Rudgers & Gardener, 2004; Sasidharan & Venkatesan, 2019; Tanaka et al., 2015; Wendel & Grover, 2015). But all these papers deal with the ecology of either EFNs or elaiosomes, even though a strong mutualistic relationship with ants is usually implicated among both. Ants are well‐known for their antiherbivore protective function in exchange for receiving extrafloral nectar (Heil, 2015; Rosumek et al., 2009; Rudgers, 2004) and separately as seed dispersers in exchange for later consumption of the nutritious elaiosome (Sasidharan & Venkatesan, 2019). This lack of integration is illustrated in one review entitled, “Current issues in the evolutionary ecology of ant–plant symbioses” that cites 18 papers about EFNs but elaiosomes are not mentioned (Mayer et al., 2014).

Despite their frequent co‐presence, the fact that they might be functionally related seems only to have been recognized in one other species, *Turneria ulmifolia*, that we could find (Cuautle et al., 2005; Dutton et al., 2016). This is a tropical vine where ants were shown to feed from the EFNs and also harvest elaiosome‐bearing seeds from the plant that enhanced seedling production. We report on a similar co‐adapted phenomenon here, but for an entirely different geographical setting: a fireprone, sclerophyll woodland in mediterranean Australia, and further, undertook in‐depth natural history observations and a rigorous test of their functional relationship, and showed that it is key to this plant's reproductive success as only plants that flower produce EFNs.

## 2 NATURAL HISTORY OBSERVATIONS

**FIGURE 1:**
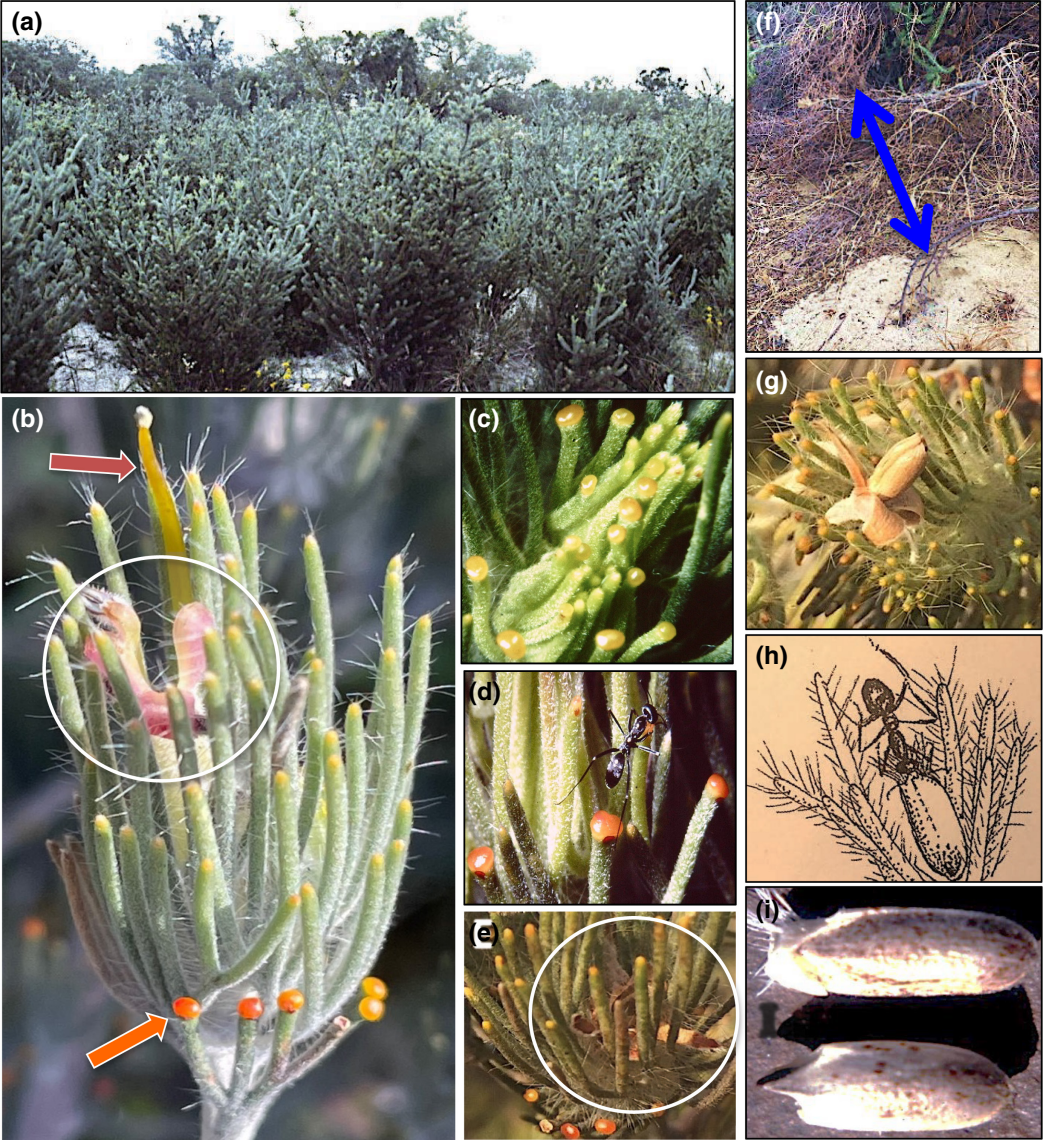
Images of plant–ant relations in the woolly bush, *Adenanthos cygnorum* (Proteaceae). (a) Thicket of woolly bush, 1.5 m tall, after clearance of woodland for highway (original woodland in background). (b) Current season's branchlet showing single flower immersed in the leaf cluster – Pink arrow indicates style bearing pollen at its tip that is transferred to bird pollinators when they probe the “gullet” of the flower (ringed) to reach nectar, orange arrow points to an EFN, one of a number that surround the leaf cluster, leaves ~25 mm long. (c) Immature new season's leaves wrapped around apex interspersed with already mature EFNs, 2 mm wide, suggestive of a protective function. (d) *Iridomyrmex bicknelli* (Formicidae), body 6 mm long, feeding at an EFN. (e) Dry bracteoles pushing leaves apart to create a leafy cup with loose seed at their base (ringed). (f) Nest of *Rhytidoponera violacea*, the major seed‐carrying ant in southwestern Australia, located 40 cm from the crown of an adult *A. cygnorum* shrub (distance indicated by blue arrow). (g) Seed, 8 mm long, held in leaf cup before breaking free from its bracteolar cup (dislodged to highlight its location relative to the seed). (h) Drawing of *Iridomyrmex bicknelli* (same species as E) removing seed from leaf cup assisted by caruncle (elaiosome). (i) Seed of *A. cygnorum* with caruncle intact (upper) and after removal by ant (lower). Photos by BBL and drawing by Susan Patrick from photo by BBL.

General observations were begun in the 1970 s throughout the range of this species in southwestern Australia (northern boundary: 27.662°S, 114.110°E to southern boundary: 33.777°S, 116.625°E, see supporting figure) and continued on an ad hoc basis for the next 40 years. The woolly bush, *Adenanthos cygnorum* (Proteaceae), grows as a thicket from soil‐stored seeds after soil disturbance or, historically more generally, fire (Figure 1a). This species occurs widely through the Swan and Eneabba coastal sandplains (climate details for both areas given in Lamont et al., 2022). It is the dominant shrub in *Banksia* woodland, yet is killed by fire in a vegetation type where 95% of the woody species survive fire, and is also prominent in disturbed and burnt sites in scrub‐heath. The region experiences a warm Mediterranean‐type climate.

Plants begin vegetative growth in the wet winter, flower in spring and produce fruits over the dry summer–autumn. Each new season's fertile branchlet commences with one or more digitate leaves wrapped around the apex with each segment terminating in a conspicuous, orange‐red EFN (Figure 1b,d,e). We observed that the nectaries start exuding on average 14 days before the branchlets produce their single open flower (Figure 1c). Ants were seen to feed from the EFNs (Figure 1e) throughout the year and quantitative details are given below. The flower is immersed in a terminal whorl of leaflets whose spaces are filled with unusually long hairs that prevent florivory and nectar‐robbing by ants (Figure 1c) that can otherwise occur among exposed flowers of similar structure in the region (Lamont, 1982). The woolly bush has a major role in maintaining resident nectar‐feeding birds (Meliphagidae) during the period when *Banksia* trees are not in flower, although seed set is only 1–3% (Newland & Wooller, 1985), possibly as a legacy of the nutrient‐impoverished sands in which this species occurs (Stock et al., 1989).

The diaspore is actually a dry indehiscent fruit fused to a single seed, about 5 mm long by 2 mm wide, that we refer to as a seed hereon. A soft, bilobed caruncle is attached to the base (Figure 1i) and, at 11.5% nitrogen (unpublished), its protein content rivals that of insect larvae (Del Valle et al., 1982). At maturity, the four to five bracteoles wrapped around the seed recurve and push apart the surrounding leaflets to produce a leafy cup. The seed then dehisces and sits loose at the base of the cup (Figure 1b). Ants were seen to harvest seeds from the cup, gripping them via the caruncle (Figure 1h), confirming that these functioned as an elaiosome, with quantitative details given below.

Husks of mature seeds were sometimes observed under the plants that may have been consumed by house mice (*Mus musculus*, Muridae); six native rodent granivores (*Pseudomys* species, Muridae) have been recorded historically in the area (Watts & Aslin, 1981). The exotic laughing dove (*Spilopelia senegalensis*, Columbidae) was a regular consumer of seeds under crowns of the study plants and, rarely, ringneck parrots (*Barnardius zonarius*, Psittaculidae) were observed consuming seeds on the plant. An endemic ground parrot (*Pezoporus flaviventris*, Psittaculidae), four quail species (*Coturnix* species, Phasianidae) and two pigeons (*Phaps chalcoptera*, *Ocyphaps lophotes*, *Columbidae*) have been recorded from the area historically and they remain active in more remote sclerophyll shrublands/woodlands elsewhere where *A. cygnorum* also occurs (Pizzey & Doyle, 1980, personal observations).

## 3 QUANTITATIVE METHODS

We quantified ant visits to plants at two study sites 15 km south of Perth, Western Australia (Jandakot Airport Reserve and a bushland remnant in Leeming, centered on 32.106°S, 115.861°E – see supporting figure). Both study sites covered 0.1 ha with *A. cygnorum* forming an open thicket in a *Banksia* woodland remnant (Figure 1a). Weekly or biweekly observations over 12 weeks were conducted through autumn–early winter in 1982 on six representative juvenile plants (lacking EFNs) and six adult plants (many EFNs on every annual branchlet, Figure 1d). Ants were identified to species, and whether they were visiting the EFNs at the time or not, at 900, 1100, 1300, 1500 and 1700 h. The number of ants observed per day was summed. Stature and width in two directions of each plant was measured and converted to volume, assuming that the shrubs were spheroidal in shape, and mean visits plus standard deviations per species per day per plant per unit crown volume determined. Data were analyzed separately for the three most common ants and means and standard errors determined over this period.

At the same time, we observed which species visited the EFNs and removed seeds and transported them down the plant. A parasitoid wasp and nine common ants were selected for more detailed observations, to include: (a) if they routinely fed at the EFNs during the study period, (b) if they were observed to transport seeds from the plants and/or removed seeds placed by us in their pathway, and (c) if they attempted to remove 10 Xylorictid moth larvae (collected from their protective shelters of webbed frass) placed on the plant. Transport of seeds to their nests was followed on three occasions. We excavated six nests (probably belonging to the most abundant harvester ant in the area, *Rhytidoponera violacea*, but uncertain as they had been abandoned) to a depth of 8 cm at the start of the wet season after a summer fire. We later unearthed 20 seedlings from the same burnt patch and estimated their depth of burial from the foot of the hypocotyl to the surface where the hypocotyl turned green (method of Lamont et al., 1993).

Since the seed is clearly nutritious and palatable, and historically highly vulnerable to a myriad of ground‐dwelling granivores, it was of interest to learn to what extent ants might serve as a granivore‐avoidance mechanism, that is, removing the seeds while on the plant before other granivores had access to them on the ground. We therefore undertook a trial to see if preventing ants from removing seeds from (a) leaf cups on the plant, or (b) beneath crowns of the plants, made a difference to the rate of seed removal by other means. Twelve isolated plants of similar size were located in the study area and 3 × 5 lots of caruncle‐bearing seeds placed under each of six plants and in the leaf cups of six plants in a haphazard fashion. Half of these in turn either had a ring of the sticky resin, tanglefoot®, placed on the ground around the edge of the crown, intended to prevent access to ants either on the ground or on the plant, or were left intact, to give four treatments in a 2 × 2 interaction design.

## 4 RESULTS AND DISCUSSION

**FIGURE 2:**
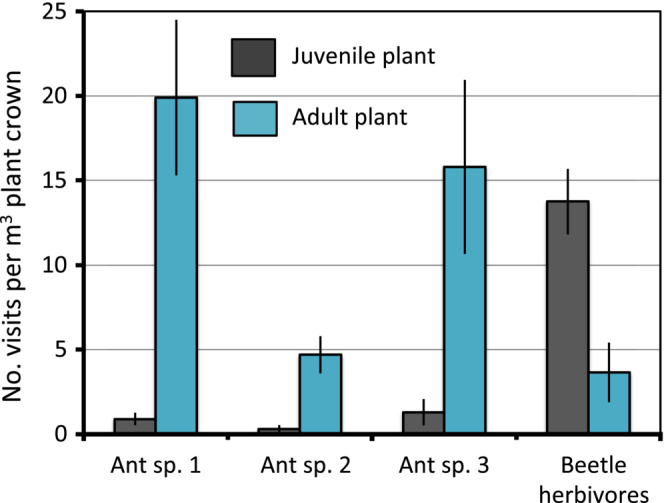
Visits by three dominant ant species to juvenile (lack EFNs) and adult (many EFNs on every branchlet) plants of *Adenanthos cygnorum* over 12 weeks in Banksia woodland, 15 km south of Perth, Western Australia. 1 = *Diceratoclinea* sp., 2 = *Polyrhachis* sp., 3 = *Rhytidoponera violacea*, and snout beetles (Curculionidae). Crown volumes of juveniles were ~ 5% that of the adults and data were corrected for this difference. Data are means plus standard errors for six plants.

**FIGURE 3:**
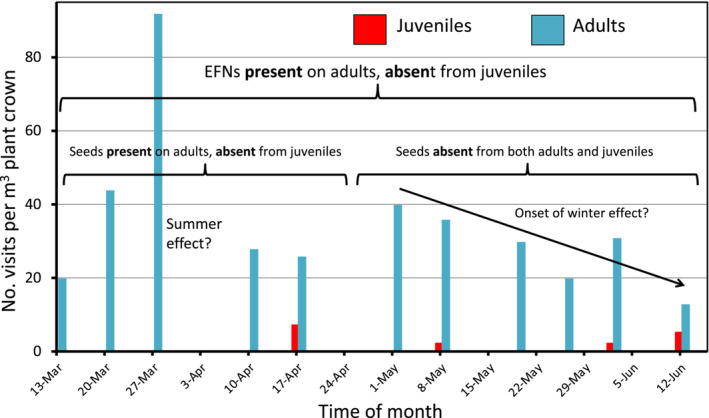
Visits by ants to juvenile and adult plants at five equally spaced times through the daylight hours in early autumn to early winter. Only ants visiting EFNs at the time of observation were scored for adult plants but not for juveniles as these lacked EFNs. While EFNs were active throughout this 3‐month period, seeds were available on the plants only for the first five visits but were absent on the following six assessments as flowering only occurred over summer. Note that the presence/absence of seeds made no clear difference to the rate of visits to the adults.

The change in leaf structure (production of EFNs on the tips of the segments, Figure 1) with the onset of maturity indicates that protection of the reproductive apices by ants is a fitness priority compared with vegetative buds that are not so protected. The protective function of the EFNs was supported on three grounds: (1) Parasitoid wasps, *Campoletis* sp. (Ichneumonidae), fed at the EFNs. They laid their eggs on the pupae of a specialist phytophagous moth, *Xylorycta* sp. (Xyloryctidae), that webs together the terminal leaves of adult branchlets and feeds on the reproductive apices (juveniles have long branches without terminal whorls so are not attractive to this herbivore). Seven of 10 pupae that we removed from their shelters hatched into *Campoletis* wasps instead of moths. (2) Visits by ants to juvenile plants, which lack EFNs, were negligible compared with the scores on adults at any time (plants were not inspected at night), even when adjusted for plant size (Figures 1e, 2, 3). That exposed Xyloryctids, or other insects, would also be attacked by at least some of the ant visitors was demonstrated by two (*Iridomyrmex conifer, Rhytidoponera* sp. 3) of nine ant species that visited the EFNs, when presented with 10 larvae, immediately grasping and carrying them down the plant (Table 1). (3) Two snout beetle herbivores (*Phylyctinus callosus*, *Naupactus leucoloma*, Curculionidae) were only abundant on the juveniles (Figure 2). This implies that their presence was deterred by some of the ant visitors to the EFN‐bearing adults by analogy with their response to moth larvae (Figure 2), although removal of eggs deposited on the leaves is another likely mechanism.

**TABLE 1:** Interaction of a parasitoid wasp and nine ant species that were frequent visitors to *Adenanthos cygnorum* (Proteaceae) shrubs with their EFNs, seeds, and Xyloryctid moth larvae in the Perth area, Western Australia

Family/subfamily	Species	Visits extrafloral nectaries	Removes seeds (seen on plant &/or on presentation of 10 seeds)	Interacts with moth larvae (on plant or on presentation of 10 larvae)
Ichneumonidae/*Campopleginae* (wasp)	*Campoletis* sp. 1	Yes	No	Parasitoid
Formicidae/Dolichoderinae (ant)	*Diceratoclinea* sp. 1	Yes	Yes	?
Formicidae/Formicinae (ant)	*Formica* sp. 1	Yes	Yes	No
Formicidae/Dolichoderinae (ant)	*Iridomyrmex agilis*	Yes	Yes	No
Formicidae/Dolichoderinae (ant)	*Iridomyrmex conifer*	Yes	Yes	Removes
Formicidae/Dolichoderinae (ant)	*Iridomyrmex bicknelli*	Yes (Figure 1)	Yes (Figure 1)	No
Formicidae/Formicinae (ant)	*Polyrhachis* sp. 1	Yes	No	No
Formicidae/Ectatomminae (ant)	*Rhytidoponera violacea*	Yes (Figure 1)	Yes (Figure 1)	No
Formicidae/Ectatomminae (ant)	*Rhytidoponera* sp. 2	Yes	Yes	No
Formicidae/Ectatomminae (ant)	*Rhytidoponera* sp. 3	Yes	No	Removes

Altogether we observed 17 ant (morpho)species and a few wasps systematically visiting the EFNs, with flies, lacewings and bugs making fleeting visits, throughout the year but especially during warm weather (Figure 3). Since annual branchlet growth occurs in discrete clusters and leaves are long‐lived, we were able to count back leaf clusters and show that EFNs could continue exuding for 4 years after which the leaves die. In one study on a warm summer day, an individual of the ant, *Rhytidoponera violacea*, passed over seven branchlets per min over 10 min, often stopping briefly at EFNs surrounding them. Since this is also the most common seed harvester ant in the region, it alludes to the second function of the EFNs: to attract seed‐harvesting ants to visit the plant on a regular basis to enhance the likelihood of seed removal.

Twelve of the 17 ant species lifted seeds, via their elaiosomes, from the leaf cups and carried them down the plant (Figure 1h). Seven of nine EFN‐visiting ant species studied more closely also removed seeds when presented with them, often cooperatively if smaller than the length of seed. One of the “pugnacious” ants also removed seeds (*Iridomyrmex conifer*) while the other (*Rhytidoponera* sp. 3) did not. Seeds were carried to nests up to 8 m away, although most nests were clustered around individual shrubs (Figure 1f), but only nests of the most common harvester ant, *Rhytidoponera violacea*, were readily identifiable to species. Excavation of six nests to a depth of 8 cm revealed 12 dormant seeds and 16 germinants, all lacking elaiosomes (Figure 1i). Unearthing 20 seedlings from a recently burnt patch showed that they arose from an average depth of 3.5 ± 1.5 (SD) cm indicating their burial in ant nests. Thus, it is reasonable to conclude that the ants not only use the elaiosome as a way to grasp the seed but also as a food source. Secondly, in so doing, not only do they not harm the seed but also their final location is consistent with providing a suitable environment for fire‐related dormancy release and germination (Pausas & Lamont, 2022).

**FIGURE 4:**
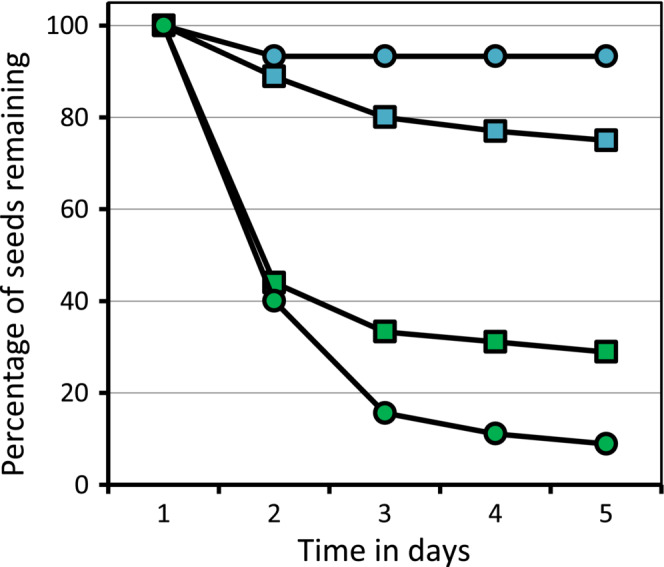
Removal of seeds of *Adenanthos cygnorum* placed in four locations over 5 days. Half the seeds were placed under the crowns (square symbols) and half were placed on terminal branchlets (circular symbols) equivalent to the position of current‐season seeds. In turn, half of each of these plants was ringed by sticky resin (tanglefoot®) (blue symbols) intended to prevent the advance of ants to where seeds were located, and half were not (green symbols) so allowing ready access by ants (green circular symbols) and vertebrate granivores as well as ants (green square symbols). Results are the sum of 15 seeds per plant for three plants per treatment. Fisher's exact probability for a treatment effect: 0.0125 (one‐tailed).

The exclusion trial also supports a third component of the EFN‐elaiosome relationship: by being retained on the plant, seeds escape granivory by ground‐dwelling vertebrates. The results were readily allocated into two groups: most (>60%) removed within 2 days when access was unfettered, and only a few (≤10%) removed within 2 days when ringed by resin (Figure 4). It is clear that the extent of ant activity allowed was solely responsible for this pattern (and that the method of ant control worked). By day 5, a further 30+% had been removed from intact plants, indicating ongoing ant activity, and none from the on‐plant resin treatment, confirming the success of the treatment in preventing ant activity. A further 20+% had been removed from the under‐plant resin treatment suggesting that some larger granivores (doves?) were able to bypass the ring to access seeds with a further 10+% from the controls, implying limited but ongoing activity of ants and other granivores. 20% more seeds were removed from the plant than from the ground (binomial probability of ≤4 seeds [as observed] remaining on the plant by chance alone out of 17 still present on and under the plants on day 5 = 0.0245, one‐tailed). Whether from habit or climbing the shrubs specifically in search of nectar, the result is increased efficiency of seed transport by ants to burial sites compared with removal from the ground not only numerically but also fate‐wise, for many of the seeds removed from the ground would be ingested (birds, rodents) rather than transported. Seeds falling to the ground cannot have contributed to the small number remaining on the plants, as only 4% of the controls were lost by day 2 (strong winds?) after which there was no further loss.

It is now clear that the co‐presence of EFNs and elaiosome‐bearing seeds forms a syndrome directed at maximizing species fitness by (1) inducing regular visits to the plant by both parasitoid wasps and aggressive and/or seed‐transporting ants, (2) attracting seed‐harvesting ants, and (3) enabling seed transport from the site of production rather than allowing them to fall to the ground where there is a greatly increased likelihood of seed removal by vertebrate granivores (Figure 4). Ants only visit mature plants as these possess EFNs, whether or not seeds are present (Figure 3), so that the primary attraction must be nectar from the EFNs. Thus, the EFNs are multifunctional, attracting both “enemies” of insect herbivores (wasps and ants) and, later, seed dispersers/buriers (sometimes the same ants). Ants visit the plants throughout the year in search of nectar as an immediate food source (for them) and opportunistically locate seeds during the warm, dry season as a later food source (for ant larvae) (Figure 3). In his world survey, Heil (2015) noted that parasitoid wasps were ranked second to ants in the list of invertebrate visitors to EFNs such that herbivore deterrence was the essence of their function. But it appears secondary in *A. cygnorum* once the central role of EFNs in seed dispersal, storage and germination via ants is recognized.

As the EFN‐elaiosome syndrome appears to enhance plant, and thus species, fitness, it is of interest to compare how it fits with various hypotheses raised about the role of myrmecochory (Giladi, 2006). Since traits that ensure removal of seeds from the plant by ants is clearly an adaptive response to strong granivore pressure, seed burial by ants is part of achieving the same end so that this must also be viewed as a priority. Nevertheless, it is difficult to rank this as more important than vertical placement in the sand that ensures receival of a suitable heat pulse during fire yet insulation from the full heat of the fire, for plants senesce in the absence of fire within 20 years and seeds left on the surface would be incinerated by the inevitable fire within this time frame. We note that seeds survive the long interval between fires (percentage survival is unknown, although none of the seeds we excavated was dead), unlike those of *Turneria ulmifolia* (see next paragraph). *A. cygnorum* does not appear to support the dispersal‐distance hypothesis (Giladi, 2006) as most nests are congregated around the host bushes (Figure 1f) that might be attributable to their being a reliable source of nectar throughout the year rather than just a seasonal supply of elaiosomes (Figure 3). Intra‐clump nest locations might support the directed‐dispersal hypothesis if it could be shown that their current location was optimal for survival rather than establishing seedlings outside the patch. At present, nest clustering seems more beneficial to the ants than it does to the plants but our observations on nest locations were anecdotal and the topic is worthy of more detailed research. Giladi (2006) also wondered if mymecochory might affect the phenology of flowering and thus seed set. In support, Lamont et al. (1985) provided clear evidence that two myrmecochorous genera in the Proteaceae, including *Adenanthos*, flowered in spring–summer rather than throughout the year as for two non‐myrmecochorous genera in the same family.

Parallels to this syndrome have only been reported for one other species so far, *Turneria ulmifolia*. The key there was (a) retention of seeds in the persistent fruits and (b) EFNs attracting a suite of ants with distinct roles: either protection or transport (Cuautle et al., 2005). That ants were predominantly seed harvesters among the woolly bush might be because (a) the species is highly sclerophyllous (Figure 1) and not particularly vulnerable to phytophagous insects, (b) a more efficient system via parasitoid wasps exists for controlling the major herbivore, *Xylorycta* sp., and (c) the threat from “large” ground‐dwelling granivores is substantial and sustained (nutrient‐rich seeds in this nutrient‐impoverished environment are at a premium). In regard to the last point, Cuautle et al. (2005) showed that seeds were more likely to be harvested from the ground than the plant, in contrast to our system (Figure 4).

Heterophylly, also confined to the reproductive phase, is also a feature of other members of the Proteaceae (*Hakea*) in the vicinity of the woolly bush. Although EFNs are not involved, the leaves produced by mature plants mimic or conceal the fruits (crypsis) to achieve similar ends although the seeds are later released in response to fire upon which they become vulnerable to granivores (Groom et al., 1994; Groom & Lamont, 2015; Lamont et al., 2016). A final similar syndrome might be recognized among *Acacia cyclops* plants that occur on coastal dunes marginal to the distribution of the woolly bush (see Figure S1). Like all acacias, it produces EFNs on the petioles throughout its lifespan but, unlike almost all others, its elaiosome is strongly convoluted and a bright orange, and the seed is retained in the open pod (Groom & Lamont, 2015). Here it is likely to be harvested by granivorous birds, but, due to its sclerotic testa, it survives digestion, that contrasts with the brittle seedcoat of *A. cygnorum*. There is no evidence that birds visit the EFNs but some bird pollinators do feed from EFNs in other species (Vanstone & Paton, 1988).

**FIGURE 5:**
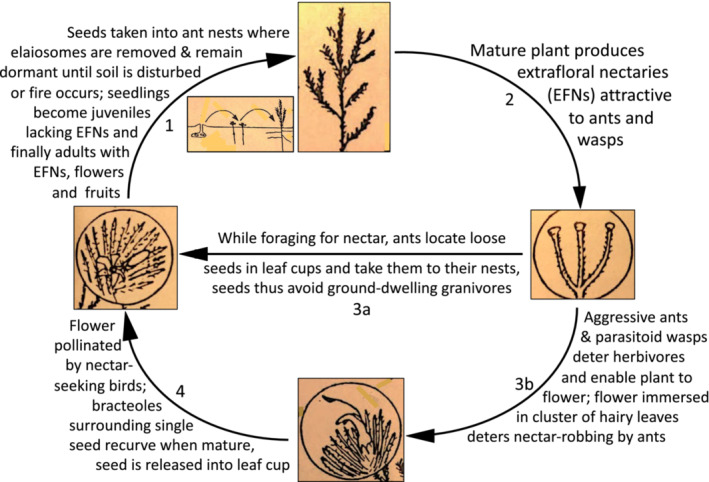
Proposed life cycle of *Adenanthos cygnorum*, with the sequence indicated by arrows beginning with 1 through to 4 (see Figure 1 for scale). Drawings and scheme prepared by BBL.

We conclude that EFNs in *Adenanthos cygnorum* are not just an indirect adaptation to minimize herbivory generally via ants, only a minority of which is aggressive (Table 1), but specifically serve to enhance reproductive success via seed‐harvesting ants (direct adaptation). Even the EFN‐visiting, parasitoid wasps serve to control moth larvae that destroy the flower‐bearing apices (indirect adaptation). The ants are offered a nectar “entrée” before receiving the “main course” of a nutrient‐rich elaiosome that ensures the seeds escape granivores and are buried. Their location at a depth of 2–5 cm is ideal for protection from granivores during storage and insulation from the full heat from fires of varying intensity, sufficient to break inherent dormancy but not too deep for the germinant to reach the surface (Pausas & Lamont, 2022). Such extraordinary efforts to ensure seeds have the maximum opportunity to produce seedlings (Figure 5) are consistent with a fireprone shrub that is killed by fire and thus must recruit after every fire, where seed set is low and probably nutrient‐limited in such nutrient‐impoverished sands (Stock et al., 1989), and where both seed‐harvesting ants and numerous vertebrate granivores are abundant. Sorting out which of these (fire, poor soils, summer drought, granivores, seed dispersers) is the key environmental constraint and thus major selective force in the evolution of this reproductive syndrome would be a considerable challenge.

There are thousands of species in numerous families spread throughout the world that possess both EFNs (or other food bodies) and elaiosome‐bearing diaspores. If ecology is to fulfill its stated role as an integrative science, then here lie many opportunities to demonstrate and consolidate that role, rather than treating the different organs of plants as acting independently of each other, as has usually been done. Quite apart from examining to what extent this phenomenon is widespread among species with different growth forms and habitat‐types throughout the world, our work must be regarded as exploratory and many aspects we raise require further investigation within this species over time, space and circumstance. For example, we paid no attention to nectar properties but note that herbivore damage my lead to increased exudation and visits by ants among some plants (Heil, 2015; Lach et al., 2009).

## AUTHOR CONTRIBUTIONS

**Byron B. Lamont:** Conceptualization (lead); formal analysis (equal); investigation (supporting); methodology (equal); project administration (lead); supervision (lead); visualization (lead); writing – original draft (lead). **James Grey:** Conceptualization (supporting); data curation (lead); formal analysis (equal); investigation (lead); methodology (supporting); resources (lead).

## FUNDING INFORMATION

This study was part of the postgraduate diploma of JG but was otherwise unfunded.

## Supporting information


Figure S1

## Data Availability

Data have been deposited in Dryad (https://doi.org/10.5061/dryad.73n5tb319).
