# Supplementary material for: Extrafloral nectar as entrée and elaiosomes as main course for ant visitors to a fireprone, mediterranean‐climate shrub
Source: Ecol Evol. 2022 Nov 8;12(11):e9500. doi: 10.1002/ece3.9500 (PMC9643124; doi:10.1002/ece3.9500)
Supplement: Supplementary file 1 — Figure S1 [file ECE3-12-e9500-s001.docx]

**
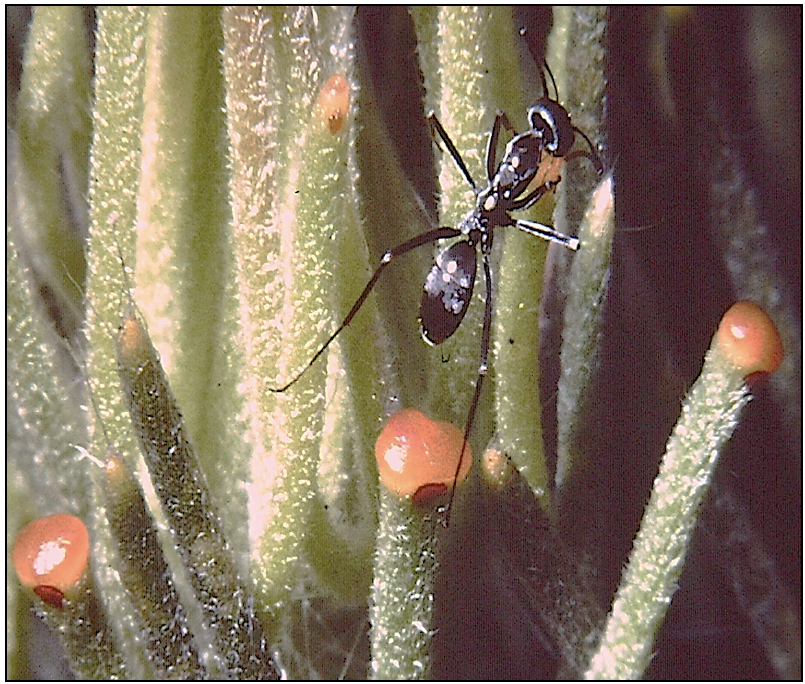
**
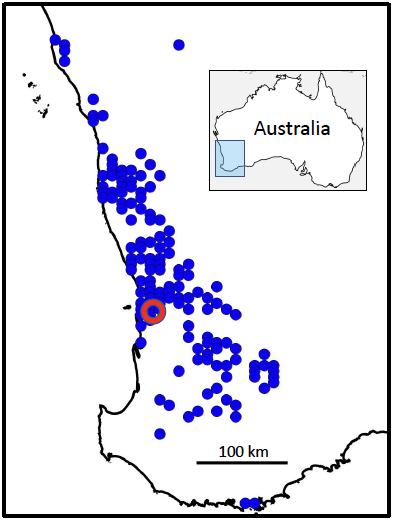


Supporting figure: Left: ant (*Iridomyrmex bicknelli*) sipping nectar from an extrafloral nectary of *Adenanthos cygnorum*, one of many such glands surrounding each flowering shoot; Right: distribution of *A. cygnorum* in the sandplains of southwestern Australia, with quantitative study area ringed. Base map downloaded from [https://avh.ala.org.au/occurrences/search?taxa=adenanthos+cygnorum](https://avh.ala.org.au/occurrences/search?taxa=adenanthos+cygnorum#tab_mapView), 25 Sept 2022. (prepared by Byron Lamont)
